# Systematic Review with Network Meta-Analysis: Antidiabetic Medication and Risk of Hepatocellular Carcinoma

**DOI:** 10.1038/srep33743

**Published:** 2016-09-19

**Authors:** Yao-Yao Zhou, Gui-Qi Zhu, Tian Liu, Ji-Na Zheng, Zhang Cheng, Tian-Tian Zou, Martin Braddock, Shen-Wen Fu, Ming-Hua Zheng

**Affiliations:** 1 Department of Cardiology, Jinhua Municipal Hospital, Jinhua 321004, China; 2 Department of Hepatology, Liver Research Center, the First Affiliated Hospital of Wenzhou Medical University, Wenzhou 325000, China; 3 School of the First Clinical Medical Sciences, Wenzhou Medical University, Wenzhou 325000, China; 4 Department of Ultrasonography, Jinhua Municipal Hospital, Jinhua 321004, China; 5 Global Medicines Development, AstraZeneca R&D, Loughborough, United Kingdom; 6 Institute of Hepatology, Wenzhou Medical University, Wenzhou 325000, China

## Abstract

Antidiabetic medication may modify the incidence of hepatocellular carcinoma (HCC). We aimed to compare the use of different antidiabetic strategies and the incidence of HCC. PubMed, Embase.com and Cochrane Library databases were searched up to 31 October 2015 and randomized controlled trials (RCTs), cohort studies or case-control studies were included for our analyses. A total of thirteen studies enrolling 481358 participants with 240678 HCC cases who received at least two different strategies were retrieved in this analysis. Direct comparisons showed that use of metformin (risk ratio [RR] 0.49, 95% CI 0.25–0.97) was associated with a significant risk reduction of HCC, while insulin (RR = 2.44, 95% CI 1.10- 5.56) may significantly increase the risk. Indirect evidence also suggested that insulin (RR = 2.37, 95% CI 1.21–4.75) was associated with a significantly increased risk of HCC. Additionally, metformin was effective in reducing the risk of HCC when compared with sulphonylurea (RR = 0.45, 95% CI 0.27–0.74) and insulin (RR = 0.28, 95% CI 0.17–0.47). Notably, metformin was hierarchically the best when compared with other antidiabetic therapies for the prevention of HCC. In summary, available evidence suggests that metformin was the most effective strategy to reduce HCC risk when compared with other antidiabetic interventions.

The incidence and mortality rate of hepatocellular carcinoma (HCC) has significantly increased over recent decades. With a poor 5-year survival rate, HCC has become the third most common cause of cancer death worldwide^1^. Although established risk factors for HCC are recognized as including hepatitis C and hepatitis B viral infection and excessive alcohol consumption, there are at least 15–50% of HCC cases without specifically recognized aetiology^2^.

Diabetes mellitus (DM) has been proposed as a potential risk factor for HCC^3^. As part of metabolic syndrome, DM is believed to share many risk factors with a variety of cancers^4^ and epidemiological evidence linking DM to HCC has been reported in recent studies around the globe^5^,^6^,^7^,^8^,^9^. The underlying mechanisms responsible for the increased risk for developing HCC in patients with DM is complex and remains a matter of debate. Emerging evidence suggests that it is likely to be related to the interplay between obesity, DM and tumorigenesis, with insulin resistance and hyperinsulinaemia playing critical roles^10^.

On this basis, recent research has suggested that the use of antidiabetic medication may modify the risk for developing HCC in several different ways^11^,^12^. Evidence from *in vitro* and *in vivo* experimental studies has shown that insulin, it’s analogs and oral insulin secretagogues (such as sulfonylureas), which may contribute to hyperinsulinemia, are suggested to increase the incidence and progression of cancer^13^. In contrast, metformin, an insulin sensitizer, not only reduces levels of circulating glucose and insulin but also has potential protective effects on carcinogenesis in patients with insulin resistance and hyperinsulinemia. Observational studies from different countries and areas support the belief that the use of antidiabetic drugs which increase insulin sensitivity, such as metformin or thiazolidinediones (TZDs), may decrease the incidence of liver cancer, while exposure to insulin and sulphonylurea has been associated with an excess HCC risk^14^,^15^,^16^. Two large randomized controlled trials (RCTs), namely, ADOPT (A Diabetes Outcome Progression Trial) and RECORD (Rosiglitazone Evaluated for Cardiovascular Outcomes and Regulation of Glycaemia in Diabetes), failed to demonstrate that metformin offered any chemopreventive effect on the development of HCC when compared with TZDs, but remained potentially advantageous over sulphonylurea^17^.

Currently, there is still no high-quality RCT assessing the potential impact of different classes of antidiabetic pharmacotherapies on modifying the risk of developing HCC in DM patients. Due to the discrepant results in the literature and limited epidemiological evidence for the precise relationship between DM treatment and the risk of HCC, this study sought to systematically review the literature to evaluate, quantify, and summarize the association between the use of different anti-diabetic medications and the development of HCC. To obtain a better understanding of this issue, we performed a network meta-analysis of available clinical studies to investigate the association between antidiabetic medications and the risk of developing HCC.

## Results

### Study characteristics

**Figure 1:**
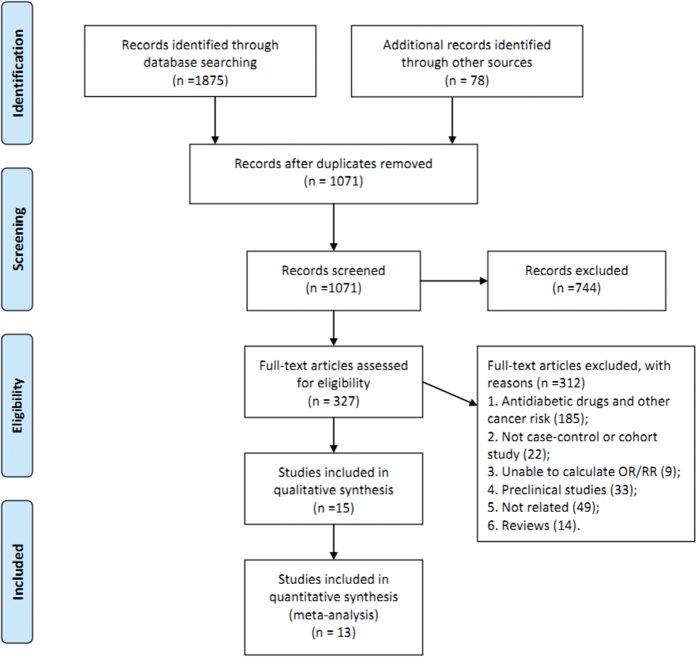
Literature search and selection.

**Figure 2:**
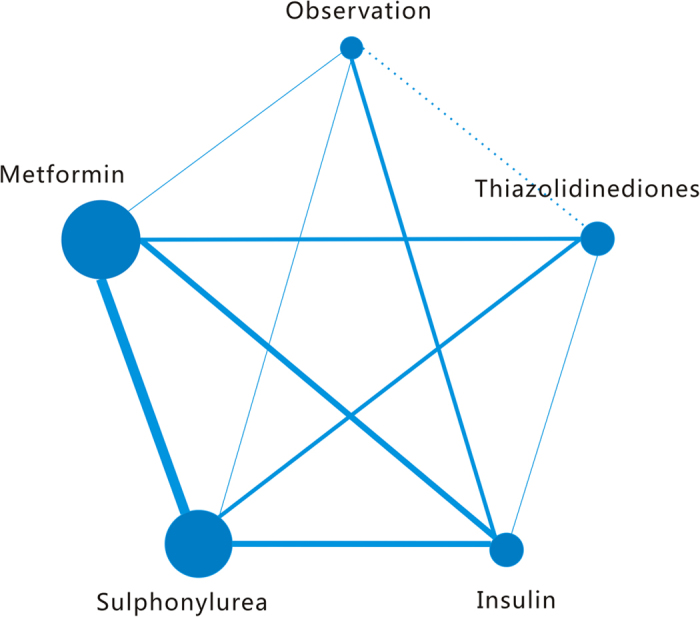
Network of the comparisons for the Bayesian network meta-analysis. The size of every node is proportional to the number of patients. The width of the lines is proportional to the number of trials or pairs of trial arms.

The participant flow diagram for study inclusion in the meta-analysis is shown in Fig. 1. A total of thirteen studies were retrieved, which were all published in English^15^,^16^,^17^,^18^,^19^,^20^,^21^,^22^,^23^,^24^,^25^,^26^,^27^. Of the 1935 potentially relevant references identified by electronic and manual searches, 744 publications were excluded according to title and abstract. After detailed assessment of the full text, a further 312 were excluded because they did not satisfy the inclusion criteria. Overall, a total of 13 studies enrolling 481358 participants with 240678 HCC cases who received at least two different treatment strategies were included in this analysis (Fig. 2), with one RCT, four cohort studies, and eight case-control studies. Table 1 summarizes the characteristics of studies which qualified for this network meta-analysis. The 13 studies were from different countries and published between 1991 and 2015. Among the 13 studies, which were mostly multiple-arm trials, patients were treated with insulin in 12 studies, metformin in 11 studies, sulphonylurea in 10 studies, and TZDs in 7 studies. Table 2 depicts the methodological quality and scores of included studies. For the cohort and case-control studies, the median score was 7 on the Newcastle–Ottawa quality assessment scale, which showed that the quality of included studies was reliable. Additionally, the quality of the RCT was assessed as moderate by the Cochrane risk of bias tool.

### Results from pair-wise comparisons

Five different comparisons including observation were performed in pairwise meta-analysis, with a lack of direct comparison between TZDs and observation alone. The weighted RRs for the occurrence of liver cancer were calculated for each comparison, the geometric distribution of which is displayed in Fig. 2. Compared with observation, meta-analysis of the direct comparisons showed that the use of metformin (RR 0.49, 95% CI 0.25–0.97) was associated with a significant risk reduction of HCC, while insulin (RR 2.44, 95% CI 1.10–5.56) appeared to increase the risk of liver cancer. Additionally, in comparisons between active interventions, metformin appeared to be significantly superior to insulin (RR 0.30, 95% CI 0.18–0.50), together with sulphonylurea (RR 0.44, 95% CI 0.27–0.72), and TZDs (RR 0.97, 95% CI 0.91–1.02), albeit with no statistical significance achieved. Despite a lack of direct comparison with observation, TZDs showed borderline significance when compared with insulin (RR 0.33, 95% CI 0.14–0.78). These results were derived from 9 independent analyses, with other comparisons failing to show results of statistical significance (Table 3).

With respect to statistical heterogeneity, it was estimated in two of the comparisons by the I^2^ statistic. Overall, statistical heterogeneity in our analysis was moderate, although for some comparisons 95% confidence intervals (CIs) were wide and included values indicating very high or no heterogeneity. As illustrated in Table 3, I^2^ values higher than 75% were recorded for 3 comparisons, that is, metformin versus insulin, metformin versus sulphonylurea, TZDs versus sulphonylurea, with remaining comparisons lower than 40%. In addition, no publication bias was found for Begg’s rank correlation test and Egger’s test among these comparisons of different antidiabetic therapies.

### Results from the network meta-analysis

**Figure 3:**
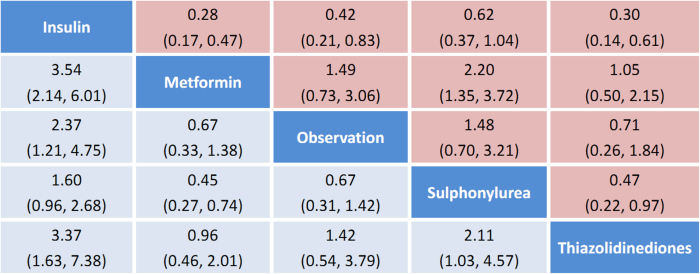
Pooled odds ratios for HCC incidence. The column treatment is compared with the row treatment. Numbers in parentheses indicate 95% confidence intervals.

We summarize the results of the random-effects network meta-analysis for HCC rates in Fig. 3, which illustrates the RRs for HCC occurrence with 95% CIs obtained from the indirect comparisons of the included regimens. Compared with observation alone, insulin (RR 2.37, 95% CI 1.21–4.75) was associated with a significant increased risk of HCC, in line with the results of direct evidence. According to network meta-analysis, metformin was significantly effective in reducing the risk of HCC when compared with sulphonylurea (RR 0.45, 95% CI 0.27–0.74) and insulin (RR 0.28, 95% CI 0.17–0.47). Likewise, TZDs showed a similar trend in more beneficial effects against HCC incidence when compared with sulphonylurea (RR 0.47, 95% CI 0.22–0.97) and insulin (RR 0.30, 95% CI 0.14–0.61).

**Figure 4:**
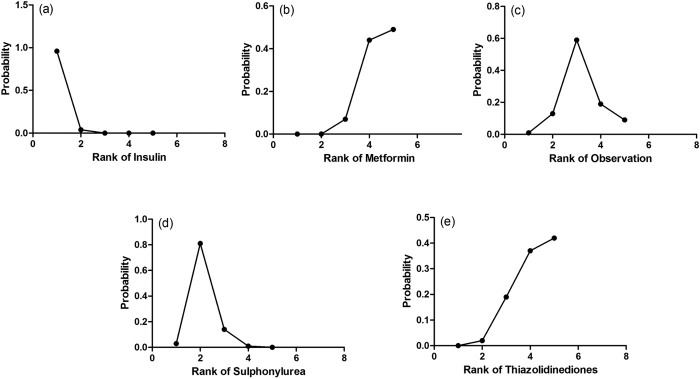
Rankograms showing probability of each strategy have each rank (1–5) for HCC incidence. Ranking indicates the probability to be the best treatment, the second best, the third best and so on. Specifically, rank 1 is worst and rank N is best.

**Figure 5:**
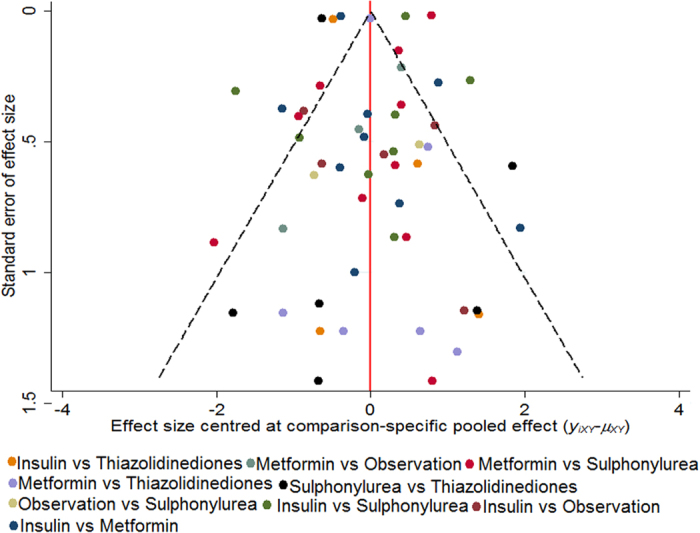
Comparison-adjusted funnel plot. The red dotted line represents the null hypothesis that the study-specific effect sizes cannot differ from the respective comparison-specific pooled effect estimates. The two black dashed lines represent a 95% CI for the difference between study-specific effect sizes and comparison-specific summary estimates. Different colors correspond to different comparisons. Yixy is the noted effect size in study i that compares x with y. μxy is the comparison-specific summary estimate for x versus y.

The probabilities of best treatment for each strategy were ranked at each of the five possible parameters (Fig. 4). In agreement with aforementioned results, metformin was hierarchically shown the best to decrease the risk of liver cancer according to the estimated surface under the cumulative curve values. Conversely, insulin was ranked the lowest for the prevention of HCC, which may suggest that it was the least effective in reducing HCC occurrence. As the comparison-adjusted funnel plot showed in Fig. 5, there was no evidence of asymmetry.

### Comparisons between traditional pairwise and network meta-analyses

According to the results of traditional pairwise (Table 3) and network meta-analyses (Fig. 3), the pooled estimates showed slight differences. The CIs from traditional pairwise meta-analyses and the Bayesian network meta-analyses in general overlapped, which suggests that the evidence derived from both methods is consistent. Overall, the P values of the node-splitting method show no significant difference between direct and indirect effects, suggesting no inconsistency within the networks for any of the 5 outcomes (Table 4).

## Discussion

This analysis, based on one RCT and 12 observational studies, showed that metformin had a chemopreventive effect on the incidence of HCC when compared with observation alone, while insulin was associated with a statistically significant increase in HCC risk. In addition, the probabilities of best treatment for each strategy suggested that metformin was the best, TZDs were the second best, sulphonylurea was the third best, and insulin was ranked the lowest in the prevention of HCC. Moreover, there was no inconsistency or publication bias in our network meta-analysis.

According to our current knowledge and available data, glucose lowering drugs can variably modify the incidence of HCC. Our finding is consistent with the current understanding that exogenous insulin therapy or insulin secretagogues may be associated with an increased incidence of hepatoma and a higher mortality because of cirrhosis and HCC^13^,^21^,^28^. As a recent meta-analysis of seven observational studies showed, insulin and sulphonylurea conferred a total of 161% and 62% increase in HCC incidence, respectively^29^. In contrast, metformin, as a first-line anti-hyperglycemic drug, has recently gained more attention for its potential to reduce cancer incidence and improve prognosis of DM patients with solid tumor^14^,^30^,^31^. In agreement with our results, DM patients taking metformin were reported to have a 76% reduction in HCC risk compared with those receiving other standard hypoglycemic therapies when data was subjected to a meta-analysis^32^. In addition, the association of exposure to metformin with a 35% reduction of cancer-related mortality was also noted^33^,^34^.

Notably, the association of DM and cancer is implicated in insulin resistance and hyperinsulineamia^10^. The administration of insulin or insulin secretogogues such as sulfonylureas, leads to exogenous or endogenous hyperinsulinemia^35^. As an important mitogen, insulin may promote cancer progression through its binding to the insulin receptor (IR) and/or IGF-1R (insulin-like growth factor receptor), which results in auto-phosphorylation of insulin-receptor substrate-1 (IRS-1) and activation of the phosphatidylinositol 3-kinase (PI3K)/Akt (serine/threonine kinase)/mammalian target of rapamycin (mTOR) pathway and the mitogen-activated protein kinase (MAPK) pathway^36^. Additionally, hyperinsulinemia increases hepatic growth hormone receptor (GHR) levels and down-regulates the level of IGF-binding protein 1, raising the bioavailablility of IGF-1 on cellular proliferation and inhibition of apoptosis^37^,^38^.

Conversely, metformin, together with other insulin sensitizers, may counteract insulin resistance and consequent hyperinsulinemia, and appears to be associated with a lower cancer risk^39^. This finding may in part be explained considering that metformin acts to inhibit hepatic gluconeogenesis, inhibits glucose uptake in the muscle and leads to an improvement of insulin sensitivity in peripheral tissue^40^,^41^. In addition, metformin may impede carcinogenesis through indirect mechanisms, which include induction of cell cycle arrest and/or apoptosis, activation of the immune system, inhibition of the unfolded protein response (UPR), leading to a possible eradication of cancer stem cells^42^. Intracellularly, metformin, when highly concentrated in the liver, may prevent protein synthesis, cell proliferation and angiogenesis through activation of AMPK (adenosine monophosphate activated protein kinase) pathway^42^,^43^. AMPK, a key mediator of tumor suppressor serine-threonine liver kinase B1 (LKB1), mechanistically serves as a cellular energy sensor essential for metabolic processes. In addition, TZDs, as peroxisome proliferator-activated receptor-γ agonists, may not only increase the sensitivity of insulin, but also trigger cell cycle arrest, apoptosis, anti-proliferative, anti-angiogenic, and pro-differentiation pathways and thus contribute to down-regulation of carcinogenesis^44^. It has been suggested that PPAR-γ activation by TZDs is able to induce an inhibitory effect on HCC metastasis. Increasing evidence from both in *vitro* and in *vivo* studies supports the concept that PPAR-γ deficiency produce an environment prone to tumorigenesis, while PPAR-γ overexpression may inhibit HCC growth. Thus, PPAR-γ could exert beneficial effects against HCC and may represent as an anti-tumorigenic and therapeutic target^45^.

Some methodologic issues should be acknowledged which could be regarded as limitations in our study. First, our findings are primarily based on data derived from observational studies, which are inevitably prone to bias and confounders. Given the retrospective or hospital-based nature of some included studies that could lead to an overestimate of effect, further prospective studies with high quality analyses are needed to validate the effect of antidiabetics on such outcomes. Secondly, details on dosage, duration of antidiabetic therapy as well as full information on other residual confounders were incomplete. Thus, the findings provided by this meta-analysis should be viewed with some caution. Thirdly, statistical heterogeneity was moderate in the meta-analyses of direct comparisons, while no substantial inconsistency was found in the network meta-analysis. The diversity in study populations, comparators, and study design is responsible for a substantial heterogeneity in effect estimates across studies. Lastly, as the number of included studies is small, a bias (including bias of indication) should be considered in interpreting the results described.

Besides these limitations, our meta-analysis has several strengths. Our study is the first Bayesian network meta-analysis performed on the potential tumor-modifying effect of antidiabetics. Based on the available literature and current knowledge, we have constructed a comprehensive and complete picture of the relationship between different antidiabetic medications and the incidence of HCC. In the absence of available studies directly comparing all eligible treatments, we have to rely on indirect comparisons of multiple treatments (such as the comparison arm of observation vs. TZDs in these studies). For instance, an indirect comparison of A with B can be made by integrating the information derived from studies of A vs. C and studies of B vs. C. Thus, the method of network meta-analysis may increase statistical power by incorporating evidence from both direct and indirect comparisons across all interventions. To ensure the accuracy and reliability of our results, this study compared the pool effect estimates of all available antidiabetic therapies, even when no head-to-head studies existed. For the included studies, major confounding factors have been controlled which include age, sex, race, socioeconomic status, body mass index, smoking, ethanol intake, hepatitis C virus infection, hepatitis B virus infection, cirrhosis, and DM duration. Data quality was reliable and the total statistical heterogeneity was moderate, with no small study effects or publication bias. Lastly, a rankogram of insulin, sulfonylureas, metformin and TZDs for chemoprevention of HCC provides a formal rank order for suggested treatment strategies for diabetic patients.

## Conclusions

In summary, our network meta-analysis provides a complete picture of the HCC-modifying effects of different antidiabetic medications by using Bayesian analytical approach. Specifically, metformin and TZDs have beneficial effects on HCC incidence, while insulin or sulphonylureas therapy may be associated with a higher risk of HCC. In light of potential confounding factors in observational studies, there remains a need for more well-designed randomized controlled trials, together with pathophysiological studies, to elucidate the potential role and the clinical efficacy of metformin and TZDs anticancer agents and to describe the details of their biological mechanism of action. Our analysis may contribute clinical decision making regarding appropriate antidiabetic treatment for patients with DM with a high risk of HCC.

## Material and Methods

### Literature Search

The protocol for this systematic review was performed in agreement with the Preferred Reporting Items of Systematic Reviews and Meta-Analyses (PRISMA) statement^46^. RCT and observational studies relevant to this meta-analysis were searched through PubMed, the Cochrane database and the EMBASE.com database, using the keywords ‘hypoglycemic agents, metformin, sulfonylurea compounds, TZDs, insulin, and cancer’ up to 31 October 2015. No language, publication date, or publication status restrictions were imposed. The most updated data for a given study or database were selected. Reference lists from cited articles were also manually searched for additional eligible trials.

### Selection criteria

Criteria for inclusion of an article in this meta-analysis were as follows: (1) RCTs and epidemiologic studies, including cohort study or case-control study; (2) the objective was designed to evaluate the relationship between hypoglycemic agents and HCC incidence; (3) patients with no prior diagnosis of primary liver cancer or other cancers most likely to metastasize to the liver; (4) risk estimates should be provided or could be estimated with sufficient information. Duplicated or overlap reports were eliminated according to the same title, author list or publication date to avoid giving double weight to estimates derived from the same research or population. We then reviewed the full articles passing the first screening of titles/abstracts.

### Data extraction

Two investigators (Zhou YY, Zhu GQ) independently reviewed the full manuscripts of eligible studies and extracted information into an electronic database: the first author, year of publication, country or area, patients’ characteristics, study design, type of hypoglycemic agents, study time or follow-up time, adjusted confounders, number of HCC cases and controls. Any discrepancies regarding the extraction of data were resolved by consensus and arbitration by an additional investigator (Zheng MH). When relevant information was unclear, or when some needed data was unavailable directly from the study, the original authors were sought for eligible data by email.

### Quality Assessment

To assess the risk of bias in the observational studies, we used the Newcastle–Ottawa Quality Assessment Scale with some modifications to match the needs of this study, which included the following items: patient selection, comparability of antidiabetic medication and observation group, and assessment of outcome. In terms of RCT, we used the Cochrane risk of bias tool to determine the quality. The quality of the methodology was independently assessed by two investigators (Zhou YY, Zhu GQ).

### Data analysis

Initially, we performed standard pairwise meta-analysis with a random-effects model by using STATA 12.0 (Stata Corporation, College Station, Texas, USA). For indirect and mixed comparisons, a Bayesian network meta-analysis followed, with a random-effect hierarchical model by means of Markov chain Monte Carlo methods with Gibbs sampling from 1,000 iterations obtained after a 5,000 iteration training phase. We performed random-effects pairwise and network meta-analyses to obtain estimates for the incidence of HCC. According to Cochrane Handbook for Systematic Reviews of Interventions Version 5.1.0, the incidence of HCC was presented as RR with 95% CIs. Clinical heterogeneity was assessed with the I^2^ statistics. A formal confirmation of heterogeneity was then obtained by referring to the I^2^ statistic, judging values of less than 25% to be minimal, 25% to 49% to be moderate, and 50% or greater to be substantial. Nevertheless, the node splitting method was also utilized to estimate inconsistency of the model, which separated evidence into direct and indirect and compared them respectively. Small study effects were explored by inspecting comparison-adjusted funnel plots. Publication bias was assessed by Begg’s rank correlation test and Egger’s test among comparisons of different antidiabetic therapies. Egger’s test and Begg’s test was then performed to evaluate publication bias by comparison of P values, which if <0.10 indicating significant publication bias.

To rank the treatments for an outcome, we estimated the relative ranking probability of each treatment and obtained the treatment hierarchy of competing interventions using rankograms, surface under the cumulative ranking probabilities. To check for the presence of inconsistency, we used the previously described node-splitting method, which separates evidence for a particular comparison into direct and indirect^47^. Specifically, the results from the traditional pairwise meta-analysis were referred to as direct evidence, while indirect evidence meant results from network meta-analysis. Then we assessed the agreement between the direct and indirect evidence and reported its Bayesian *P* value.

## Additional Information

**How to cite this article**: Zhou, Y.-Y. *et al.* Systematic Review with Network Meta-Analysis: Antidiabetic Medication and Risk of Hepatocellular Carcinoma. *Sci. Rep.* **6**, 33743; doi: 10.1038/srep33743 (2016).

## Figures and Tables

**Table 1:** Characteristics of included studies.

Studies	Design	Location	Study population	Study period	Mean follow-up (years)	Cases defined	Total no. of subjects	No. of HCC cases	Adjusted confounders^a^	Antidiabetic types^b^
Yu *et al.*^18^	Case-control	USA	Cases of newly diagnosed hepatocellular carcinoma in Los Angeles County and controls from the neighborhood	1984–1990	NR	histologically confirmed	32	18	1–4, 7–10, 13	I
Oliveria *et al.*^19^	Cohort	USA	Diabetes patients	2000–2004	3.9	ICD-9	16705	9	4, 5, 7–10	M,T,S,I
Donadon *et al.*^15^	Case-control	Italy	Diabetes patients with chronic liver disease	1994–2008	NR	NR	549	190	1, 2, 5, 7–9	M,S,I
Hassan *et al.*^20^	Case-control	USA	Cases of newly diagnosed HCC in the Cancer Center and controls from genetically unrelated family.	2000–2008	NR	NR	217	124	1–4, 6–9	M,T,S,I
Home *et al.*^17^	RCT (ADOPT)	UK	Diabetes patients	2000–2006	4	NR	4351	4	1–6, 13	M,T,S,I
Home *et al.*^17^	RCT (RECORD)	UK	Diabetes patients	2001–2008	5.5	NR	2225	2	1–6, 13	M,T,S,I
Home *et al.*^17^	RCT (RECORD)	UK	Diabetes patients	2001–2008	5.5	NR	2222	2	1–6, 13	M,T,S,I
Kawaguchi *et al.*^21^	Nested case-control	Japan	Hepatitis C patients with diabetes mellitus	2004–2008	NR	NR	143	96	1, 2, 5, 7, 10	M,T,S,I
Nkontchou *et al.*^22^	Cohort	France	Diabetes patients	1988–2007	5	histologically confirmed or noninvasive criteria	100	39	1, 2, 4, 5, 9, 12, 13	M,S,I
Chang *et al.*^16^	Nested case-control	Taiwan	Diabetes patients	2000	NR	ICD-9	115183	25236	1, 2, 4, 14	M,T,S,I
Ruiter *et al.*^23^	Cohort	Netherlands	All individuals with prescription for any hypoglycemic drug based on hospital record database	1998–2008	2.8	ICD-9	85289	31	1, 2, 14	M,S
Schlesinger *et al.*^24^	Cohort	UK	General people	1992–2000	8.5	ICD-10	8324	2089	1, 2, 4–7, 13	I
Hagberg *et al.*^25^	Nested case-control	USA	Diabetes patients	1988–2011	NR	Read codes B150300, B150z00, and B152.00	690	121	1, 2, 5–9, 14	M,I
Bosetti *et al.*^26^	Nested case-control	Italy	Diabetes patients	2005–2007	6	ICD-9	4477	209	1, 2, 14	M,S,I
Miele *et al.*^27^	Case-control	Italy	General people	2005–2012	NR	AASLD guidelines	171	102	1, 2, 6, 7	M,S,I

^a^1, age; 2, sex; 3, race; 4, socioeconomic status; 5, body mass index; 6, smoking; 7, ethanol intake; 8, HBV infection; 9, HCV infection; 10, cirrhosis; 11, alcoholic liver disease; 12, on-alcoholic liver disease; 13, diabetes mellitus duration; 14. medications taken (unspecified).

^b^M: Metformin, T: Thiazolidinediones, S: Sulphonylurea, I: Insulin.

**Table 2:** Quality assessment of included studies.

Studies	Observational studies^a^								
	Selection				Comparability	Outcome or exposure			Score
	1	2	3	4	5	6	7	8	
Yu *et al.*^18^	*	*	*	*	**	*			*******
Oliveria *et al.*^19^	*	*	*	*	**	*	*	*	*********
Donadon *et al.*^15^	*	*		*	**	*		*	*******
Hassan *et al.*^20^	*	*		*	**	*		*	*******
Kawaguchi *et al.*^21^	*	*		*	**	*		*	*******
Nkontchou *et al.*^22^	*	*			**	*		*	******
Chang *et al.*^16^	*	*	*	*	**	*		*	********
Ruiter *et al.*^23^	*	*		*	**	*	*	*	********
Schlesinger *et al.*^24^	*	*		*	**	*	*	*	********
Hagberg *et al.*^25^	*	*	*	*	**	*		*	********
Bosetti *et al.*^26^	*	*	*	*	**	*		*	********
Miele *et al.*^27^	*	*		*	**	*		*	*******
Randomized clinical trial^b^									
	1	2	3	4	5	6	7		
Home *et al.*^17^	L	L	L	L	H	U	L		

^a^The quality of the observational studies was performed using the Newcastle–Ottawa Quality Assessment Scale.

^b^The quality of the RCT was assessed by Cochrane risk of bias tool (L = low risk, H = high risk, U = unclear risk). 1: random sequence generation; 2: allocation concealment; 3: blinding of participants and researchers; 4: blinding of outcome assessment; 5: incomplete outcome data; 6: selective reporting; 7: other bias.

**Table 3:** Outcomes and assessment of heterogeneity and publication bias in traditional meta-analysis.

Treatment comparisons	Results of pair-wise meta-analysis	H (I^2^%)	P values of Begg’s test	P values of Egger’s test
M vs I	0.30 (0.18, 0.50)	38.92 (79.4)	0.81	0.57
S vs I	0.68 (0.46, 1.02)	27.81 (74.8)	0.62	0.28
T vs I	0.33 (0.14, 0.78)	6.12 (51.0)	1	0.28
I vs O	2.44 (1.10, 5.56)	10.97 (63.5)	0.62	0.45
M vs O	0.49 (0.25, 0.97)	3.78 (47.1)	0.11	0.11
M vs S	0.44 (0.27, 0.72)	64.12 (86.0)	0.25	0.01
M vs T	0.97 (0.91, 1.02)	4.12 (0)	0.19	0.24
S vs O	1.30 (0.34, 4.97)	2.85 (64.9)	1	NA
T vs S	0.50 (0.15, 1.68)	21.52 (76.8)	0.57	0.91

Notes: NA: not available; O: Observation; M: Metformin; T: Thiazolidinediones; S: Sulphonylurea; I: Insulin.

**Table 4:** Asseessment of inconsistency between direct and indirect evidence.

Treatment comparisons	Direct effect	Indirect effect	Overall	*P*-Value of Node-Splitting Method
I vs M	−1.24 (−1.79, −0.72)	−0.88 (−2.17, 0.34)	−1.26 (−1.79, −0.76)	0.57
I vs S	−0.34 (−0.84, 0.16)	−1.19 (2.38, −0.00)	−0.47 (−0.99, 0.44)	0.18
I vs T	−1.23 (−2.17, −0.37)	−0.51 (−2.11, 1.03)	−1.21 (−2.00, −0.49)	0.43
M vs O	0.68 (−0.13, 1.54)	−0.75 (−2.13, 0.65)	0.40 (−0.32, 1.12)	0.07
M vs S	0.78 (0.33, 1.27)	−0.03 (−3.93, 3.62)	0.79 (0.30, 1.31)	0.65
M vs T	−0.19 (−0.94, 0.57)	0.78 (−3.02, 4.84)	0.05 (−0.70, 0.77)	0.58
O vs S	0.38 (−0.88, 1.63)	0.29 (−0.57, 1.10)	0.39 (−0.35, 1.17)	0.89
S vs T	−0.68 (−1.54, 0.12)	−1.69 (−5.22, 1.31)	−0.75 (−1.52, −0.03)	0.52

Notes: O: Observation, M: Metformin, T: Thiazolidinediones, S: Sulphonylurea, I: Insulin.
